# Epigenetics applied to child and adolescent mental health: Progress, challenges and opportunities

**DOI:** 10.1002/jcv2.12133

**Published:** 2022-12-23

**Authors:** Charlotte A. M. Cecil, Alexander Neumann, Esther Walton

**Affiliations:** ^1^ Department of Child and Adolescent Psychiatry/Psychology Erasmus MC‐Sophia Rotterdam The Netherlands; ^2^ Department of Epidemiology Erasmus MC Rotterdam The Netherlands; ^3^ Department of Biomedical Data Sciences Molecular Epidemiology Leiden University Medical Center Leiden The Netherlands; ^4^ Complex Genetics of Alzheimer's Disease Group VIB Center for Molecular Neurology VIB Antwerp Belgium; ^5^ Department of Biomedical Sciences University of Antwerp Antwerp Belgium; ^6^ Department of Psychology University of Bath Bath UK

**Keywords:** child and adolescent psychopathology, development, DNA methylation, epigenetics, population

## Abstract

**Background:**

Epigenetic processes are fast emerging as a promising molecular system in the search for both biomarkers and mechanisms underlying human health and disease risk, including psychopathology.

**Methods:**

In this review, we discuss the application of epigenetics (specifically DNA methylation) to research in child and adolescent mental health, with a focus on the use of developmentally sensitive datasets, such as prospective, population‐based cohorts. We look back at lessons learned to date, highlight current developments in the field and areas of priority for future research. We also reflect on why epigenetic research on child and adolescent mental health currently lags behind other areas of epigenetic research and what we can do to overcome existing barriers.

**Results:**

To move the field forward, we advocate for the need of large‐scale, harmonized, collaborative efforts that explicitly account for the time‐varying nature of epigenetic and mental health data across development.

**Conclusion:**

We conclude with a perspective on what the future may hold in terms of translational applications as more robust signals emerge from epigenetic research on child and adolescent mental health.

## Graphical abstract

In this paper, we discuss the application of epigenetics to research in child and adolescent mental health. We look back at lessons learned, highlight current developments in the field and point to areas of priority for future research. We also reflect on why epigenetic research on child and adolescent mental health currently lags behind other areas of epigenetic research and what we can do to overcome existing barriers, before concluding with a view on potential translational applications that could emerge as the field matures.

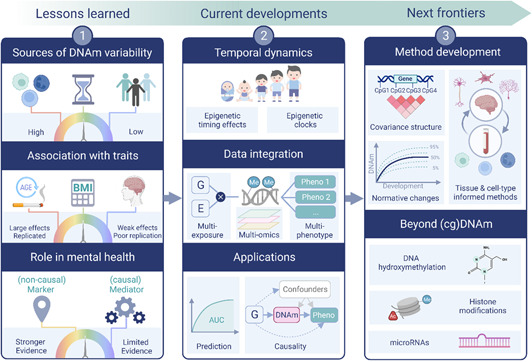

**Key points**

- Epigenetic processes, such as DNA methylation, are a promising molecular system for understanding complex gene‐environment‐development interactions on mental health.
- Despite tremendous growth in the field of psychiatric epigenetics, research focussed on child and adolescent mental health continues to lag behind.
- Closing this gap will require a shift towards better‐powered, harmonized, multi‐cohort studies that can adequately capture the time‐varying nature of DNA methylation and mental health.
- Bringing ‘timing’ at the forefront of epigenetic research can enhance prediction and mechanistic understanding of child and adolescent psychopathology.

## INTRODUCTION

Half of mental illnesses are established before the age of 18 years, often manifesting first in childhood as emotional, behavioural and neurodevelopmental problems (Solmi et al., 2022). This points to early life as a critical window of opportunity for timely detection, prevention and intervention. Although numerous pre‐ and postnatal risk factors have already been identified (e.g., parental psychopathology, socio‐economic hardship, childhood adversities; Barker et al., 2018), associations with child and adolescent mental health outcomes are far from straightforward, with equifinality (i.e., multiple risks associating with the same outcome) and multifinality (the same risk factor associating with multiple outcomes) representing the norm rather than the exception. This leaves the puzzling question of how, exactly, different mental health problems can emerge from what is seemingly a plethora of common, non‐specific risk factors. The answer to this question is largely thought to lie in the way that exposures interact with other important factors, such as a child's genetic predispositions and developmental status (i.e., timing of exposure in relation to a child's maturation level), leading to growing calls for integrative research that considers complex gene‐environment‐development dynamics (GED; Boyce et al., 2020).

Epigenetic processes involved in gene regulation represent a particularly attractive biological system for studying GED interplay. Of these, DNA methylation (DNAm) is currently the most widely investigated and best understood epigenetic process, as it is relatively easy and cost‐effective to quantify on a large scale. DNAm involves the addition of methyl molecules to DNA base pairs, typically in the context of cytosine‐guanine (CpG) dinucleotides. Studies have shown that DNAm: (1) is partly under genetic control (Min et al., 2021); (2) is sensitive to environmental influences beginning in utero (e.g., dietary, chemical and psychosocial exposures (Cowley et al., 2018; González‐Peña et al., 2021; Rijlaarsdam et al., 2017)); (3) is temporally dynamic, playing an essential role in (neuro)development (Mulder et al., 2020); and that (4) aberrations in DNAm associate with a wide range of health outcomes, including psychiatric disorders (Liu et al., 2018). As a result, DNAm has gained much interest in the search for both biomarkers and mechanisms underlying GED interplay on psychopathology. In this review, we discuss some of the complexities and unique opportunities of studying DNAm in the context of child and adolescent mental health. We provide readers with a view on major lessons learned, current developments, and emerging topics in this rapidly growing field. Considering how understudied this area is relative to, for example, epigenetics applied to adult health, we borrow some of the concepts and findings from other research areas, reflecting on potential implications for child and adolescent mental health. We conclude with recommendations for moving the field forward and a perspective on what the future may hold in terms of translational applications for research and clinical practice in child and adolescent mental health.

## LOOKING BACK: LESSONS LEARNED

Human epigenetic research has seen tremendous growth over recent years. Here, we describe three key lessons that we have learned from this research, and what implications they have for the application of DNAm to child and adolescent mental health (Figure 1.1).

**FIGURE 1:**
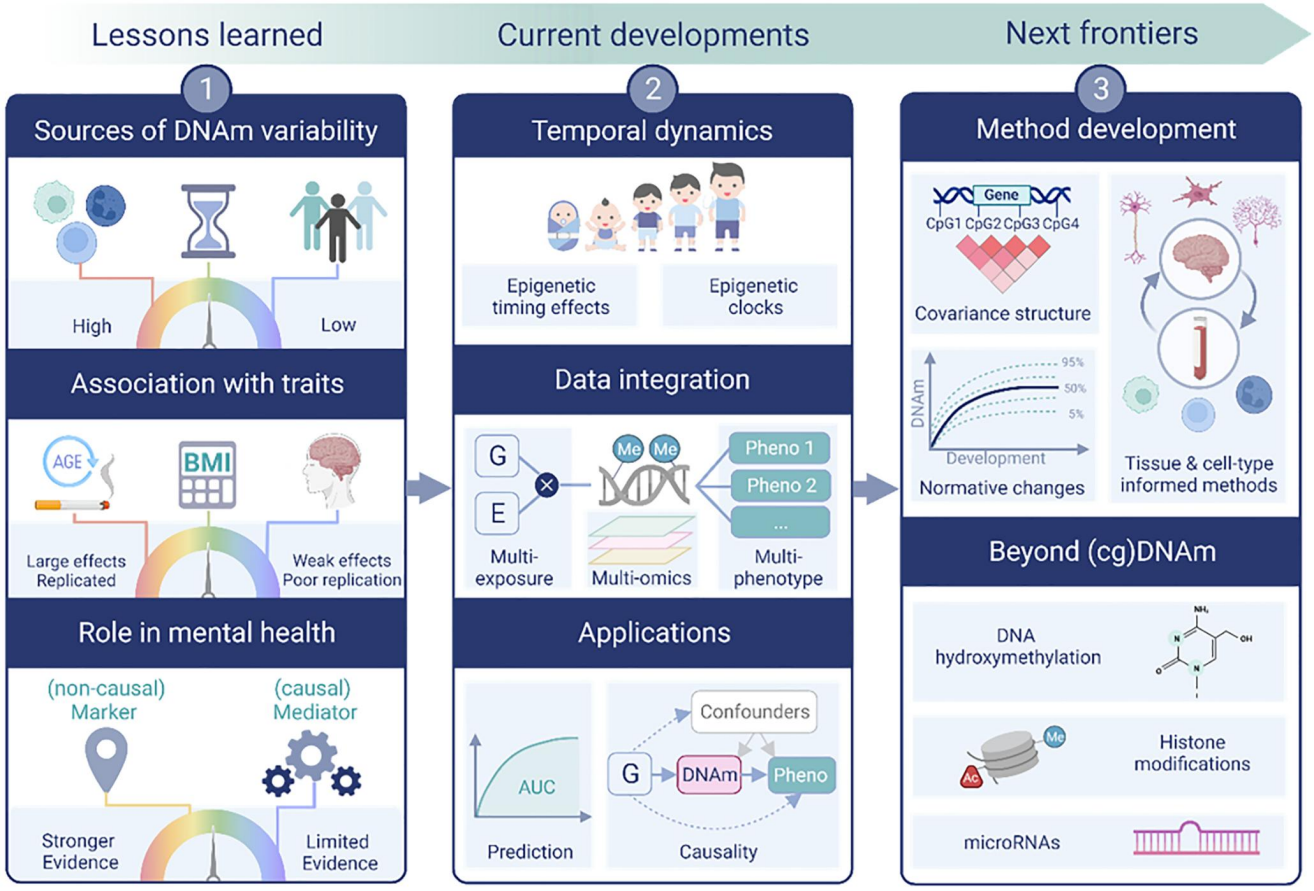
Graphical overview of themes discussed in this review. Created with BioRender.com

### Ways in which DNAm does and does not vary

One of the main lessons we have learned from existing research is that DNAm is a highly dynamic process, and that different factors contribute to this variability. Topping the list of factors is arguably *tissue and cell‐type*: for most DNAm sites, cross‐tissue variability exceeds that of inter‐individual variability within the same tissue (Hannon et al., 2015). This is particularly consequential for the study of brain‐based phenotypes (e.g., mental health traits), given that DNAm measured from easily accessible tissues (e.g., blood) may show limited correspondence to those in the target tissue of interest (i.e., brain; Bakulski et al., 2016). Even within the brain, DNAm patterns can vary between different regions and cell‐types (Edgar et al., 2017; Rizzardi et al., 2019). Another important factor is *time*: in a recent study pooling longitudinal, epigenome‐wide data from over 2000 individuals, we found that more than half of DNAm sites change significantly during the first two decades of life, and can do so in a non‐linear way (i.e., at different rates across development; Mulder et al., 2020). By comparison, studies indicate only *modest variability across individuals* for many measured DNAm sites, leading to a continuing debate about the usefulness of including seemingly non‐variable sites in analyses. For example, DNAm patterns in many regions remain static (e.g., those related to cell‐type differentiation and identity), and it is estimated that only about 20% of all methylation sites are variable (Ziller et al., 2013).

#### How is this shaping where we are going?

This evidence highlights the importance of: (1) considering cross‐tissue variability and evaluating whether peripheral‐brain concordance is important for the research question examined (e.g., concordance may be less relevant in the search for biomarkers compared to mechanistic research); (2) carefully choosing the timing of DNAm assessment and taking into account (non‐linear) temporal changes in DNAm, as these could suggest developmentally‐sensitive periods; and (3) better characterizing DNAm sites at a population level to define what should be considered (biologically) meaningful variability, with potential implications for how arrays are designed in future (Gunasekara et al., 2019).

### Many associations, few replicated

One of the strongest and most robust associations to emerge from population‐based epigenetic studies is that of DNAm with smoking exposure. The largest epigenome‐wide association study (EWAS) meta‐analysis to date has identified over 5000 significant DNAm sites in relation to prenatal smoking exposure in newborns (9 cohorts; *N*_pooled_ = 5648), and over 35,000 sites in relation to own smoking in adults (16 cohorts; *N*_pooled_ = 15,907), with hypo‐methylation of the DNAm site cg05575921 (annotated to the *AHRR* gene) showing the most pronounced difference across studies (Sikdar et al., 2019). Other traits that show widespread and replicated associations with DNAm include (gestational) age and (birth) weight (Küpers et al., 2019; Merid et al., 2020). The picture is starkly different in the realm of mental health. While many associations have been reported in the literature, these typically show much smaller effects, are derived from single datasets of modest sample size and mostly await replication. Reproducibility in mental health research has been particularly hampered by heterogeneity in study characteristics, choice of assessments and methodology, adjustment for important confounders (e.g., medication use), the temporally dynamic (and often episodic) nature of many mental health phenotypes, as well as uneven uptake of open science practices, although standards are constantly improving (Walton et al., 2020).

#### How is this shaping where we are going?

Following in the footsteps of genetic research, several strategies are increasingly being used to address study heterogeneity and small effect sizes, including: (1) establishing and leveraging collaborative initiatives to maximize power, generalizability and comparability between studies (while being mindful to retain data quality and depth of phenotyping); (2) embedding replication efforts to identify reliable markers; (3) using repositories to make analysis protocols and results fully available to improve transparency and reusability of research findings (e.g., EWAS Atlas and EWAS catalogue; Battram et al., 2022; Li et al., 2019); and (4) moving from a focus on single DNAm sites to aggregate scores (also referred to methylation risk scores [MRS]) that capture broader DNAm signatures associated with a phenotype of interest (Hüls & Czamara, 2020). Recent work from the Pregnancy and Childhood Epigenetics (PACE) consortium (Felix et al., 2018)—comprising over 40 paediatric cohorts with DNAm data at 1+ time points—is powerfully illustrating how these strategies can be applied to study risk factors (Sammallahti et al., 2021) and outcomes (Caramaschi et al., 2022; Neumann et al., 2020) related to child and adolescent mental health.

### Peripheral DNAm: Growing support as a biological marker; mixed evidence as a mediator

Early interest in DNAm in the context of mental health was largely focussed on its potential role as a mediator of environmental (and more recently also genetic) influences on psychiatric outcomes. However, increased awareness of the ‘tissue issue’ has cast more doubt on the biological plausibility of peripheral DNAm as a mechanism underlying psychiatric risk. While experimental models have evidenced multiple ways in which DNAm in blood and brain tissue can be linked (Walton et al., 2019, 2020), it remains difficult to evaluate these models in humans. Soberingly, a recent large‐scale study applying Mendelian randomization found limited evidence for a causal role of blood‐based DNAm in neuropsychiatric disorders (Min et al., 2021).

Although cross‐tissue variability makes mechanistic discoveries challenging, it does not undercut the potential of DNAm as a biological marker for disease prediction, stratification and diagnosis. Indeed, peripheral DNAm patterns are already being used to estimate a range of exposures, traits and health outcomes (e.g., age, smoking, BMI; McCartney et al., 2018) based on algorithms trained from large datasets, and to detect certain diseases sooner and more accurately than conventional diagnostic methods, leading to improved clinical care (Chen, Zang, et al., 2020; Priesterbach‐Ackley et al., 2020). These applications have been slower to permeate the field of mental health, likely due to the more limited availability of sufficiently powered datasets and challenges with psychiatric phenotypes, such as heterogeneity in clinical presentation and assessment approaches. Nevertheless, the prospect of methylation‐based profiling of neurodevelopmental and psychiatric conditions appears increasingly possible.

#### How is this shaping where we are going?

Large, longitudinal datasets and advanced methods are needed to disentangle the directionality and causality of associations between peripheral DNAm patterns and mental health outcomes. In this context, developmental research is all the more important. Even if associations are not causal and unlikely intervention targets, DNAm can still have utility as a biomarker or as a proxy for causal processes, thereby (indirectly) lending mechanistic insights.

## CURRENT DEVELOPMENTS IN THE FIELD: HIGHLIGHTS

In this section, we highlight research areas that are gaining increasing traction and lending new insights into the relationship between DNAm and (mental) health. Where possible, we refer specifically to findings on child and adolescent psychopathology, but also note research in adjacent fields that could be applied within a developmental context in the future (Figure 1.2).

### Epigenetic timing effects on neurodevelopmental outcomes

While still rare, the increased availability of birth cohorts with repeated epigenetic data in the same individuals has recently made it possible to explore key developmental aspects of the relationship between DNAm and mental health, including (1) whether DNAm patterns measured at birth (i.e. *before symptom onset*) associate with mental health problems later on; and (2) whether these associations remain *stable or change* across time. Findings have been intriguing, showing that DNAm profiles at birth (cord blood) associate more strongly with certain neurodevelopmental problems, particularly ADHD symptoms, than DNAm measured cross‐sectionally during childhood (whole blood)—a ‘timing effect’ initially observed in single cohorts (Walton et al., 2017) and recently confirmed via multi‐cohort meta‐analysis (Neumann et al., 2020). Top DNAm sites at birth implicate, among others, genes involved in neural functions (e.g., myelination, neurotransmitter release). The most notable example is *ST3GAL3*: common variation in this gene has also been identified as a top GWAS hit for ADHD (Demontis et al., 2019; Klein et al., 2019), rare mutations of *ST3GAL3* associate with cognitive and motor developmental delays (Khamirani et al., 2021), and *ST3GAL3* knockout in mice results in profound cognitive deficits and hyperactivity due to myelination disruption (Rivero et al., 2021). Similar epigenetic timing effects (i.e., where prospective associations at birth show overall a stronger signal in EWAS results than cross‐sectional associations in childhood) have also been observed for other neurodevelopmental phenotypes (e.g., social communication deficits (Rijlaarsdam et al., 2021)), but *not* for broader child mental (e.g., general psychopathology (Rijlaarsdam et al., 2022), sleep problems (Sammallahti et al., 2022)) or physical (e.g., BMI; Vehmeijer et al., 2020) health outcomes, despite studies using largely overlapping data, which points to a degree of phenotypic specificity. A detailed overview of epigenetic timing effects in the context of neurodevelopmental conditions such as ADHD, outstanding questions and research priorities in this area can be found elsewhere (Cecil & Nigg, 2022).

Why is the discovery of epigenetic timing effects meaningful? In addition to highlighting the dynamic nature of associations between DNAm and neurodevelopmental outcomes, this finding has two major implications: (1) it supports the potential of DNAm as an early pre‐symptomatic marker of neurodevelopmental risk, and (2) it suggests that, to benefit from this potential marker, the timing of DNAm assessment could be crucial—the DNAm risk signal captured at birth may no longer be detectable when DNAm is measured later in life (Walton, 2019). Timing effects may also explain some of the seemingly inconsistent findings in the literature, as studies have sampled DNAm at widely different ages. Advanced approaches capable of handling large‐scale epigenetic data at repeated time points (e.g., structured life‐course modelling, structural equation modelling, and time‐course analyses (Brown et al., 2020; Dunn et al., 2019; Hill et al., 2019; Simons et al., 2017)) are needed to further characterize and disentangle these timing effects, although separating true temporal signals from technical sources of variation in longitudinal data (e.g., batch effects) will be challenging. In future, studies will also need to establish whether a signal similar to the one observed in cord blood could be obtained from other neonatal peripheral tissues, such as neonatal blood spots, which are routinely collected from heel pricks during the first week of life in many countries and are already widely used for screening and diagnostic purposes. Ultimately, epigenetic signals at birth could inform strategies for improved early risk detection (e.g., by integrating DNAm markers in multi‐modal assessment tools including other known risk factors), and shed light on biological correlates underlying neurodevelopmental risk.

### Differentiating between biological and chronological age: Epigenetic clocks

Recent years have seen a surge of interest in the concept of biological ageing and its links to health. In the context of DNAm, a number of ‘epigenetic clocks’ have been developed that can predict chronological age (Horvath & Raj, 2018), the pace of ageing (Belsky et al., 2020) or declining health and mortality (Lu et al., 2019). In adults, epigenetic age acceleration (i.e., residual or differences scores, where DNAm‐estimated age outpaces chronological age) has been associated with a myriad of factors, including socio‐demographic characteristics (e.g., male sex, low socio‐economic status), unhealthy behaviours (e.g., smoking, alcohol use), poor health outcomes (e.g., obesity, cancer, heart disease), all‐cause mortality and, less consistently, brain outcomes (e.g., brain health, schizophrenia and depression, lower cognitive ability, total brain volume, cortical thinning, and greater vascular lesions in old age (Oblak et al., 2021)). For a comprehensive review of the applications of DNAm clocks, related challenges and recommendations, see Bell et al. (2019).

In contrast to adult studies, the application and significance of epigenetic clocks during development is far less clear. One challenge is methodological: most clocks are trained primarily on adult samples using wide age ranges and show less accuracy in paediatric samples (Sanders et al., 2022). While certain clocks have been specifically developed in paediatric samples, these focus either on cord blood to estimate gestational age at birth, or peripheral blood/buccal cells in childhood and adolescence, complicating efforts to characterize and integrate epigenetic age measures across these stages of development (Wang & Zhou, 2021). A second challenge is more conceptual: while being ‘epigenetically’ older than one's age is an indicator of risk in later life, this may not necessarily be the case during development. For example, low gestational age at birth (indicating developmental immaturity) is a known risk factor for poor mental and physical health outcomes (Aarnoudse‐Moens et al., 2009; den Dekker et al., 2016; Eves et al., 2021). In line with this, preliminary evidence suggests that age *deceleration* at birth associates with prenatal environmental adversities (e.g., maternal depression) and offspring internalizing problems later on (Suarez et al., 2018); whereas age *acceleration* in childhood and adolescence relates to postnatal environmental adversities (e.g., trauma exposure) and depressive symptoms (Sumner et al., 2019). It is important to note, however, that findings so far are based on individual studies of modest sample size, and currently lack independent replication. This, together with findings that DNAm levels can change at different rates across development, and between different tissues, adds a further layer of complexity to the study of epigenetic ageing in early life (Dieckmann et al., 2021). A promising avenue is the use of repeated measures of DNAm from birth onward to estimate a *pace of development* clock (i.e., focussing on *intra‐*as opposed to *inter*‐individual change in epigenetic age) as well as to map dynamic associations between risk exposures, epigenetic clocks and child outcomes as they unfold across different developmental stages.

### Integration on multiple levels

As the field matures, it is becoming increasingly clear that we need to move towards integrative research to better capture the complexity of DNAm and its relationship to mental health.

#### Gene‐environment (G‐E) integration

Current research mainly examines genetic or environmental effects on DNAm separately. This is problematic as environmental exposures may be genetically confounded, and in turn, genetic effects on DNAm may be environmentally modulated (i.e., potentially actionable). Consideration of both G and E is thus essential to precisely identify influences on DNAm. This is supported by evidence that variation in DNAm is best explained by *joint* (i.e., additive and interactive) G‐E effects, rather than G or E alone (Czamara et al., 2019, 2021). G‐E integration can be achieved in several ways, ranging from database queries allowing one to estimate genetic effects on DNAm sites of interest when genotyping is not available (e.g., GoDMC: http://mqtldb.godmc.org.uk/; Min et al., 2021); currently limited to DNAm sites on the 450k array), to the use of polygenic risk scores (PRS; e.g., as confounders or moderators of E effects) when genotyping is available but sample sizes are modest. Ideally, studies would integrate genetic and environmental data directly; however, this will require access to sufficiently powered datasets. To add complexity, G‐E influences should be examined and interpreted in the context of developmental timing (in line with the GED framework of psychopathology; Boyce et al., 2020), as their effects can vary with age. This is well‐exemplified by a recent study showing that glucocorticoid exposure during the proliferation stage of neural progenitor hippocampal cells, but not during post‐differentiation, alters DNAm patterns causing long‐lasting changes in the cells' response to future stressors (i.e., ‘priming’ effect; Provençal et al., 2020). In another study, timing of childhood adversity was found to explain more variability in DNAm than alternative models (e.g., accumulation or recency of exposure), pointing again to the importance of developmentally‐sensitive epigenetic research (Dunn et al., 2019).

#### Multi‐omics and multi‐phenotype integration

The extent to which statistically significant DNAm sites are also *functionally* relevant is often unclear. To address these challenges, studies have begun to integrate multiple layers of biological data. Most commonly, this involves the use of transcriptomic data to test whether DNAm sites of interest associate with gene expression levels, either measured directly in peripheral tissues (with potentially limited relevance to the brain), or examined indirectly in the brain through the use of openly accessible resources. While many of these resources exist to help researchers functionally annotate and characterize findings (e.g., GTEx for gene expression (The GTEX Consortium, 2020); GoDMC for genetic effects on DNAm (Min et al., 2021); blood‐brain comparison tools for cross‐tissue concordance (Edgar et al., 2017)), these often lack sample context and cell‐type specific resolution. Another promising type of data integration, which is helping to clarify links between peripheral DNAm patterns and the brain in vivo, is the combination of DNAm and neuroimaging (Walton et al., 2020)—although this only provides an indirect measure of peripheral‐brain associations rather than a tool for directly inferring functional effects of DNAm on the brain. Besides lending biological insights, multi‐omics integration may also help to achieve more powerful predictive models, as effect sizes from psychiatric EWASs are generally small, suggesting that DNAm patterns alone are likely to explain only limited variance in these phenotypes. Going forward, multivariate approaches will also be needed to account for the known co‐occurrence of both (1) risk factors for psychopathology (e.g., parental psychopathology and childhood maltreatment); and (2) different domains of psychopathology themselves (e.g., internalizing and externalizing problems). In this respect, methods that are already established in the field of genetics, such as multi‐trait GWAS (Wu et al., 2020) or genomic SEM (Grotzinger et al., 2019), could be extended for use with DNAm data.

### DNAm in prediction

One of the key interests in DNAm lies in its potential as a predictor of health and disease risk. Indeed, the concept of ‘methylation‐based health profiling’ has gained increased traction in recent years and is already demonstrating some success. For example, tools such as *MethylDetectR* (Hillary & Marioni, 2021) enable users to estimate a range of human traits (e.g., age, BMI), lifestyle characteristics (e.g., smoking and alcohol use) and biochemical variables (e.g., neurological and inflammatory proteins) based on peripheral DNAm alone. These DNAm‐based estimates have a number of potential advantages: they allow users to obtain information on data that is not directly available in their dataset (e.g., proteomics; Gadd et al., 2022), they may offer more reliable information on certain variables (e.g., smoking) than more bias‐prone traditional assessments (e.g., self‐report; Bojesen et al., 2017), and importantly may perform better as predictors of disease risk. For example, DNAm‐based estimates of BMI in adults have been found to predict risk for diabetes more strongly than BMI itself (Wahl et al., 2017). Although less mature, the application of predictive models to psychiatric epigenetics is beginning to bear fruit. For example, adult studies have reported reproducible blood‐based DNAm ‘signatures’ of suicide risk (Clive et al., 2016), schizophrenia(Chen, Zang, et al., 2020), and future depression risk, showing greater explanatory power than models using genetic or clinical data alone (Clark et al., 2020). Furthermore, a large EWAS study in adults found that DNAm patterns in blood explain a substantial proportion of variance in general cognitive function (*g*), and that a methylation‐based predictor derived from these results performs similarly to measured cognitive ability in predicting outcomes in independent samples, as well as generalizing across different age ranges and peripheral tissues (McCartney et al., 2022; Raffington et al., 2021). Another study employing a sequencing‐based approach recently identified a DNAm signature of trauma exposure in early adolescence, which predicted future psychiatric and health outcomes more strongly than self‐reported trauma (van den Oord et al., 2022). Importantly, the majority of these predictive DNAm sites were no longer associated with outcomes when measured again in adulthood, which provides further support for the temporally‐dynamic nature of DNAm‐mental health associations. It is noteworthy that area under the curve (AUC) estimates in these studies are not far off from well‐established, clinically implemented predictive models, such as the Framingham Risk Score used to predict coronary heart disease (Tzoulaki et al., 2009). Whether similar prediction can be achieved for child and adolescent mental health outcomes is currently unclear. In the future, it would be interesting to see how well cord blood DNAm performs in predictive models of neurodevelopmental problems such as ADHD (e.g., compared to baseline models using more established risk factors), given the observed epigenetic timing effects described above.

### DNAm and causal inference

The past years have seen major developments in the application of epidemiological methods to epigenetic research (Adams, 2019; Yousefi et al., 2022), leading to an increased appreciation of the challenges faced in making causal inferences about the role of peripheral DNAm on (mental) health outcomes. These include (genetic) confounding, reverse causation, biological constraints (e.g., tissue and cell‐type specificity) and more general methodological issues such as missing data and representativeness. Of note, most studies on DNAm and mental health are restricted to participants of European ancestry. This limits the generalizability of findings and risks accentuating health disparities, as future precision medicine tools based on epigenetic data may disproportionately benefit populations from which they were developed, underscoring the need for more diverse samples. Approaches that are being used to overcome the above challenges include the use of prospective data, repeated measures to model directionality of associations, better control for known and unknown confounders (e.g., via surrogate variable analysis), and the application of more advanced causal inference analyses leveraging genetic data, such as Mendelian randomization (MR). In the context of epigenetics, MR typically involves the use of mQTLs as genetic proxies for a particular DNAm site of interest (i.e., the ‘exposure’), to test its effect on an outcome while minimizing the potential for confounding and reverse causality. Using this method, studies have shown, for example, that DNAm patterns in blood are more likely a consequence of than a cause for BMI, consistent with findings from longitudinal observational data (Reed et al., 2020). So far, very few epigenetic studies have applied MR to child and adolescent brain‐based phenotypes. One EWAS of seizures across development used MR to test the directionality of associations, finding that seizures might be causal for changes in methylation in blood, rather than vice‐versa (Caramaschi et al., 2020). An extension of MR (2‐Step MR; Relton & Davey Smith, 2012) further allows to examine the role of peripheral DNAm as a potential casual *mediator* of environmental exposures on outcomes of interest. Using this method, a study found support for cord blood DNAm as a causal link between maternal vitamin B12 during pregnancy and offspring later cognitive function (Caramaschi et al., 2017, p. 12). As more well‐powered mQTL and EWAS studies continue to emerge, MR will become an increasingly feasible and attractive approach for strengthening causality in epigenetic research on mental health. Other strategies that could be used in future to triangulate evidence include the use of negative controls (e.g., comparing the effect of paternal vs. maternal prenatal exposures on offspring DNAm and downstream outcomes when examining in utero effects), novel techniques for modelling epigenome‐wide mediation (e.g., Divide‐Aggregate Composite null Test, DACT; Liu et al., 2020) as well as trio genetic data to better parse genetically versus environmentally‐mediated effects on the epigenome and mental health.

## NEXT FRONTIERS

What is on the horizon for epigenetic research on (child and adolescent) mental health? To push the boundaries of what is currently possible, we first need to reach a fuller understanding of epigenetic data itself (Figure 1.3).

### Mapping the covariance structure of DNAm

One property of DNAm that we still know little about is its ‘*internal structure’* (i.e., patterns of covariance). The field of population genetics has made great strides in defining linkage disequilibrium (LD) in genetic data, enabling key developments such as the imputation of genome‐wide data from a limited set of measured SNPs, improved polygenic score analyses as well as genetic heritability and correlation estimations based on summary statistics (Allegrini et al., 2022; Bulik‐Sullivan et al., 2015). Mapping the covariance structure of DNAm would similarly open possibilities to adapt these methods to epigenetic data and propel the field forward. For example, the ability to impute unmeasured DNAm sites would not only allow one to gain more information from currently available arrays, but also facilitate pooling of results from samples using different arrays, as routinely done in genetic studies. The feasibility of this endeavour, however, is uncertain. On the one hand, DNAm sites that show a more consistent covariance may be under the strongest genetic control, and thus of limited relevance for capturing exposure‐related DNAm patterns. On the other, more dynamic sites may be influenced by a range of time‐varying, individual‐level factors (e.g., cell‐type, age, environmental, biological and disease‐related factors) that could affect the covariance structure of DNAm, making it difficult to predict.

### Normative modelling and the rise of ‘chronoepigenetics’

The *time‐varying nature* of DNAm is another property that must be better characterized. As mentioned in previous sections, longitudinal studies have identified epigenetic timing effects on neurodevelopmental outcomes, while research on epigenetic clocks points to the dynamic nature of associations between risk exposures, biological age and health outcomes. Together, this evidence highlights the importance of bringing ‘timing’ at the forefront of the epigenetic research agenda, particularly for fields concerned with developmental questions, as in the case of child and adolescent mental health. Going forward, this will mean investing in longitudinal epigenetic data spanning (pre)birth to adulthood. For example, birth cohorts such as ALSPAC (UK) and the Generation R Study (the Netherlands) are continuing to expand their epigenetic resource through large‐scale profiling of DNAm patterns at four or more time points across development. Such resources could be used to generate normative models (as done in paediatrics [i.e., growth charts] and increasingly in neuroimaging (Bethlehem et al., 2022; Marquand et al., 2019)), to estimate the degree to which DNAm levels in an individual at a given time point deviate from normative developmental curves. This knowledge could also be used to weigh DNAm sites in cross‐sectional analyses based on information regarding their known pattern of stability or change over time. At a more granular level, there is increased awareness that DNAm patterns can vary throughout the day based on circadian rhythm, resulting in cyclic epigenetic oscillations (Oh & Petronis, 2021). Although disruptions in these cycles have been implicated in ageing and disease risk, little is known about their association with mental health outcomes, pointing to an interesting avenue for future research.

### New ways of addressing the ‘tissue issue’

Clearly, *tissue and cell‐type heterogeneity* in DNAm remains a major challenge for epigenetic research, and we will need to keep improving the ways we take this heterogeneity into account from study design (e.g., biomarker vs. mechanistic research) to data analysis and interpretation. Current studies typically rely on algorithms to estimate and adjust for cell‐type proportions. These algorithms, however, are imperfect and account for a limited set of cell‐types. In future, such panels could be expanded, enabling us to better capture developmental changes in cell‐type composition (e.g., including multipotent cells found in cord blood at birth, but scarcely present in peripheral blood later in life), as well as to extend recent methods for performing cell‐type specific EWAS from bulk tissue (Rahmani et al., 2019). It will also be important to evaluate whether prior knowledge of cross‐tissue concordance may be used to improve signal in psychiatric epigenetic studies, for example, by selecting or weighing DNAm sites based on blood‐brain correlations, or prioritizing regions that show high inter‐individual variability in combination with low cross‐tissue variability (Gunasekara et al., 2019).

### Moving beyond (cg) DNAm

Finally, it is important to note that (cg) DNAm is only one of multiple types of epigenetic factors, which likely play a role in neurodevelopment and show potential as markers or mediators of psychiatric risk. These include other types of DNAm marks found to be enriched in the brain (e.g., hydroxymethylcytosine; Spiers et al., 2017) as well as histone modifications implicated in several neurodevelopmental and psychiatric conditions, including autism spectrum disorder (Tseng et al., 2022). Further, experimental studies increasingly point to circulating microRNAs as a potential mechanism underlying intergenerational transmission of phenotypes, including stress‐related physiological and behavioural alterations (Lempradl, 2020), while population‐based studies in adults (Mens et al., 2021) are beginning to reveal their potential as biomarkers of disease. Currently, these types of data are still rare in paediatric studies. In future, large‐scale profiling of multiple epigenetic marks during development will be needed to characterize their independent and joint contribution to child and adolescent mental health.

## TAKING STOCK: WHY IS THE APPLICATION OF EPIGENETICS TO CHILD AND ADOLESCENT MENTAL HEALTH LAGGING BEHIND?

**FIGURE 2:**
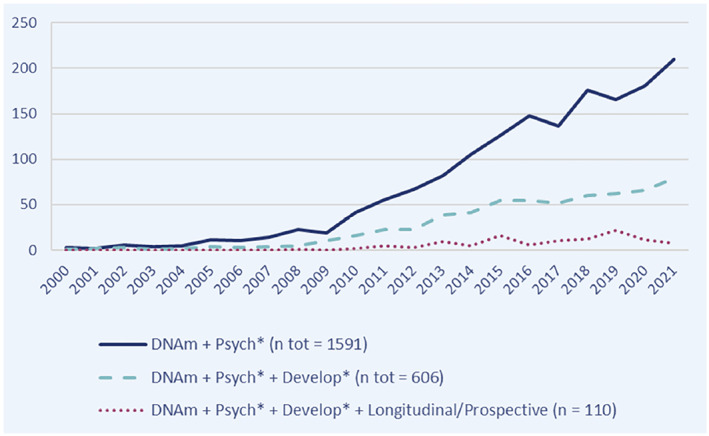
Trends in psychiatric epigenetics: Number of published DNAm studies (2000–2021). Scopus search (access date: 6th May 2022)

As readers will have likely noticed, many of the new findings and developments highlighted in this review do not originate directly from the field of child and adolescent mental health, but rather from fields adjacent to it. This reflects a broader trend in the literature: despite tremendous growth in psychiatric (and more broadly health‐related) epigenetics over the past 2 decades, the application of DNAm to child and adolescent mental health continues to account for only a fraction of this work (see Figure 2). Paradoxically, most psychiatric disorders have developmental origins, making the period between pregnancy and young adulthood arguably the most relevant, at least from the perspective of risk prediction and aetiological understanding. So, what is behind this research gap?

### Challenges

Success in epigenetic research applied to complex traits has largely rested on the ability to carry out well‐designed and adequately powered studies, usually involving multi‐cohort collaboration, to detect robust associations. Although great strides have been made in this direction, achieving the same standard for research on child and adolescent mental health has been especially challenging for two main reasons: (1) the *stage of life* and (2) the *phenotypes* under consideration. Development is characterized by more rapid and drastic biopsychosocial changes compared to adulthood, making it a particularly interesting, but also difficult, period of life to study. To capture these changes, paediatric data needs to be collected more frequently and at closer intervals than adult studies, implying more administrative costs and an increased commitment from families. Given limited resources, paediatric cohorts must typically select key time points to focus on, which may miss important developmental changes and often do not overlap (completely) between cohorts. While this problem is not exclusive to paediatric studies, the implications are likely greater: for example, adult data collected at 30 versus 35 years of age between cohorts is likely to be more comparable than paediatric data collected at birth versus 5 years, which clearly tap into different developmental periods. Together, the dynamic nature of development, combined with the availability of data at non‐overlapping time points across studies, complicates harmonization efforts needed to achieve adequate power to robustly detect subtle associations. This time of life also comes with additional ethical safeguards, which can have an impact on the choice of whether to even collect biological material, and which tissue to select. Indeed, while (longitudinal) population‐based paediatric cohorts typically collect blood, (cross‐sectional) high‐risk and clinical paediatric studies more commonly assess DNAm from less invasive peripheral tissues (e.g., saliva, buccal samples), which further limits opportunities to bridge these complementary study types. The second challenge relates to mental health phenotypes themselves. Their characteristics, even in adulthood, make them difficult targets to study (e.g., their clinical heterogeneity, low specificity and ‘fuzzy’ diagnostic boundaries) compared to phenotypes that have shown more success in EWAS studies, and which are easier to measure objectively (e.g., BMI, hip‐to‐waist ratio). Developmental research has an added layer of complexity, as psychiatric phenotypes are more dynamic in early life (e.g., differing in age of onset and temporal course), and there is less consensus on how best to measure them (e.g., type of assessment tool and rater, including parents, teachers or based on self‐reports), contributing to study heterogeneity.

### Recommendations

What can we do to address current challenges and stimulate epigenetic research on child and adolescent mental health? With the goal of harmonization in mind, it will be important to reach a better consensus on the time points and intervals of importance within this area of research. Previous studies have highlighted the ‘first 1000 days’ as a key window for (neuro)development (Cusick & Georgieff, 2016), while adolescence has also been identified by many as a critical time point (e.g., due to factors such as pubertal development). Such periods could be prioritized to maximize comparability between studies, and where possible supplemented by more fine‐grained and study‐specific time points of data collection. This goal can be achieved largely through better use of existing data. Many studies have collected, but not yet processed, biospecimens at different time points (sometimes stored for years or decades), which could be epigenotyped to capture these developmental periods and maximize correspondence with other cohorts. Researchers could also consider linking data from large biobanks and medical records, in line with legal and ethical standards. For example, many hospitals routinely collect heel‐prick samples from newborns, which could be linked with electronic health data gathered across development and used for research purposes. Relatedly, optimal choice of peripheral tissue will rest on gaining a better understanding of whether certain tissues are more informative for a given phenotype (e.g., blood for capturing immune‐related processes vs. the use of saliva/buccal samples to target cells originating from the same ectodermal germ layer as the brain), as well as careful consideration their respective drawbacks (e.g. blood collection being more invasive, while saliva and buccal samples require restricted eating/drinking prior to collection). Success in this field will also depend on improving outcome definition and harmonization of mental health phenotypes across studies. Consortia are helping to lead the way in this respect. For example, the LifeCycle project (Jaddoe et al., 2020) has undertaken a massive effort to harmonize environmental, biological and (mental) health data across paediatric cohorts including over 250,000 children as part of their EU Child Cohort Network (Nader et al., 2021). The PACE consortium has further helped to set standards for EWAS meta‐analyses involving paediatric data (Felix et al., 2018). Finally, several new initiatives, including the ADHD‐Epigenetics Working Group of the Psychiatric Genetics Consortium (PGC) and the EU‐funded TEMPO project have been established to tackle the complexity of timing in epigenetic research on child and adolescent mental health, in order to improve harmonization of epigenetic data during development and advance capabilities for longitudinal modelling of DNAm‐mental health associations.

## A LOOK TO THE FUTURE: TRANSLATIONAL APPLICATIONS

As the field matures and more robust epigenetic associations emerge, the question of how this information may be used clinically becomes increasingly relevant. To address this question, genetic research may give us useful insights into where we may be heading.

Translational applications of genetic research findings have focussed to a large degree on PRSs. With ever increasing sample sizes, the variance explained by PRSs are improving steadily. For instance, a PRS of height based on more than five million participants explained about 40% of variance and 80% of its SNP heritability (Yengo et al., 2022). Many child and adolescent psychiatric disorders are more heritable than height, but PRSs generally explain less than 5% of their variance (Jansen et al., 2020; Li & He, 2021). This can likely be explained by the much lower sample sizes (typically under 100,000 participants) and higher measurement errors of the discovery GWASs. The low variance explained casts doubt on the clinical utility of current psychiatric PRSs. However, some have argued that they may already be sufficient to identify extreme cases, aid in differential diagnosis and improve treatment response (Fullerton & Nurnberger, 2019).

Could adding information on DNAm bring these applications a step closer to clinical practice? In addition to common variants, DNAm may capture genetic effects that are not measured by SNP arrays, such as rare variants, as well as gene‐environment correlations and interactions. Furthermore, DNAm may act as a ‘biological record’ of environmental exposures, leading to more reliable assessments (e.g., cg05575921 methylation vs. self‐reported smoking) and greater predictive power compared to alternative measurement approaches (e.g., MRS of trauma exposure predicting psychiatric risk better than self‐reported trauma). Unlike genetic data, the time‐varying nature of DNAm also offers possibilities (and unique challenges) for tracking disease status and health over time, which could be particularly useful for early risk detection, patient stratification and response to treatment. In this respect, there is already much interest in utilizing epigenetic clocks in adulthood as markers of healthy ageing, which may be extended earlier in life to evaluate healthy development. Whereas these epigenetic clocks may be used as broad health markers; they may not fully capture disease‐specific pathological mechanisms. Epigenetic predictors trained on specific outcomes could provide more nuanced information about particular health profiles to inform diagnoses and guide decision‐making in more concrete clinical settings. Regarding diagnosis, it is notable that tools relying on ‘epi‐signatures’ from peripheral blood have already been developed for a wide range of Mendelian neurodevelopmental diseases, demonstrating utility for brain‐based disorders (Aref‐Eshghi et al., 2020). Whether epigenetic‐based tools could one day be used to improve diagnostic accuracy of child and adolescent psychiatric conditions—and whether the benefits of such tools would outweigh potential risks and ethical concerns—is an important topic for future research.

At the same time, MRS development will likely face the same challenge of insufficient discovery sample sizes as for PRSs in the past. Individual effect sizes of DNAm sites are not appreciably larger than SNP effects, but sample sizes of EWAS are many magnitudes lower compared to GWAS. Furthermore, unlike GWAS, we have yet to reach a consensus regarding the use of standardized pipelines for pre‐processing of DNAm arrays, including which method to choose for data normalization and batch correction—an important step for maximising comparability between studies and reducing noise due to technical variation in EWAS meta‐analyses. As mentioned in previous sections, while it is possible to impute unmeasured SNPs from genotyping arrays, this is more challenging for DNAm arrays. As such, EWAS studies and potential downstream applications, including MRS development, are confined to measured probes, which represent only a small fraction of DNAm sites on the genome. Lastly, reverse causality may limit the application of cross‐sectional data to develop methylation‐based predictive tools, and confounding may provide misleading therapeutic targets.

In conclusion, the field is still in its infancy, and concrete translational applications remain a distant goal. Nevertheless, DNAm continues to hold unique potential as a biological system for biomarker discovery and mechanistic insights into the aetiology of child and adolescent psychiatric disorders. Looking to the future, increases in sample sizes—via collaborative science, harmonization efforts and better use of existing data—in combination with a focus on developmentally‐sensitive, longitudinal study designs will be crucial to move the field forward and leverage this potential.

## AUTHOR CONTRIBUTIONS

**Charlotte A. M. Cecil**: Conceptualization, Funding acquisition, Project administration, Supervision, Visualization, Writing—original draft, Writing – review & editing. **Alexander Neumann:** Conceptualization, Writing—original draft, Writing—review & editing. **Esther Walton:** Conceptualization, Writing—original draft, Writing—review & editing.

## CONFLICT OF INTEREST

The authors have declared that they have no competing or potential conflicts of interest.

## ETHICAL CONSIDERATIONS

Not applicable.

## Data Availability

Data sharing not applicable to this article as no datasets were generated or analysed during the current study.
